# Medical resource utilization and costs for intraoperative and early postoperative periprosthetic hip fractures following total hip arthroplasty in the medicare population: A retrospective cohort study

**DOI:** 10.1097/MD.0000000000015986

**Published:** 2019-06-21

**Authors:** Abhishek Shirish Chitnis, Jack Mantel, Mollie Vanderkarr, Matthew Putnam, Jill Ruppenkamp, Chantal Elisabeth Holy, Joshua Bridgens

**Affiliations:** a Real World Data Sciences, Medical Devices, Epidemiology, Johnson & Johnson, New Brunswick, NJ; b Health Economics and Market Access, DePuy Synthes, Leeds, UK; c Health Economics and Market Access, DePuy Synthes, West Chester, PA; d Trauma, CMF, Biomaterials, DePuy Synthes West Chester, PA; VAMC, Minneapolis, MN; US Army; e Medical Affairs, DePuy Synthes, Leeds, UK.

**Keywords:** costs, early postoperative periprosthetic hip fractures, intraoperative periprosthetic hip fractures, medical resource utilization, medicare, total hip arthroplasty

## Abstract

This study assessed the impact of intraoperative and early postoperative periprosthetic hip fractures (PPHFx) after primary total hip arthroplasty (THA) on health care resource utilization and costs in the Medicare population.

This retrospective observational cohort study used health care claims from the United States Centers for Medicare and Medicaid Standard Analytic File (100%) sample. Patients aged 65+ with primary THA between 2010 and 2016 were identified and divided into 3 groups – patients with intraoperative PPHFx, patients with postoperative PPHFx within 90 days of THA, and patients without PPHFx. A multi-level matching technique, using direct and propensity score matching was used. The proportion of patients admitted at least once to skilled nursing facility (SNF), inpatient rehabilitation facility (IRF), and readmission during the 0 to 90 or 0 to 365 day period after THA as well as the total all-cause payments during those periods were compared between patients in PPHFx groups and patients without PPHFx.

After dual matching, a total 4460 patients for intraoperative and 2658 patients for postoperative PPHFx analyses were included. Utilization of any 90-day post-acute services was statistically significantly higher among patients in both PPHFx groups versus those without PPHFx: for intraoperative analysis, SNF (41.7% vs 30.8%), IRF (17.7% vs 10.1%), and readmissions (17.6% vs 11.5%); for postoperative analysis, SNF (64.5% vs 28.7%), IRF (22.6% vs 7.2%), and readmissions (92.8% vs 8.8%) (all *P* < .0001). The mean 90-day total all-cause payments were significantly higher in both intraoperative ($30,114 vs $21,229) and postoperative ($53,669 vs $ 19,817, *P* < .0001) PPHFx groups versus those without PPHFx. All trends were similar in the 365-day follow up.

Patients with intraoperative and early postoperative PPHFx had statistically significantly higher resource utilization and payments than patients without PPHFx after primary THA. The differences observed during the 90-day follow up were continued over the 1-year period as well.

## 1 Introduction

Total hip arthroplasty (THA) is recognized to be an effective procedure for treating pain and restoring mobility in patients with hip joint pathology. The effectiveness of THA has led to increasing numbers of procedures being undertaken. In the United States, the overall frequency for THA has risen over the course of recent decades and it is projected to reach an annual incidence of 572,000 by 2030.^[1]^

Periprosthetic hip fracture (PPHFx) is a major and devastating complication which can occur in patients who have undergone THA. It is associated with an increase in other postoperative complications, which may lead to a worse clinical outcome,^[2]^ as well as an increased mortality rate. Recent literature suggests an 11% 1-year mortality rate after PPHFx, pointing out that age and type of surgery are the potential risk factors.^[3,4]^ Increased life expectancy combined with the increased numbers of arthroplasties is contributing to an increase in the number of PPHFx in the United States and worldwide.^[4]^

PPHFx is categorized into intraoperative and postoperative fractures. Intraoperative PPHFx occur during the course of the initial THA procedure, while the postoperative PPHFx occur after the initial THA procedure, most commonly within the first month postoperatively.^[5]^ In a meta-analysis, Sidler-Maier et al determined that the incidence of PPHFx ranges from 0.1% to 27.8% for intraoperative and from 0.07% to 18% for postoperative PPHFx.^[6,7]^ The prevalence of intraoperative PPHFx has increased in recent years due to the increased use of cementless press-fit implants, while the increase in postoperative PPHFx appears associated with the overall increase of the at-risk population undergoing arthroplasty.^[8,9]^ In terms of treatment, the postoperative PPHFx require complicated and serious reoperations while recognized intraoperative PPHFx can be treated during primary surgery causing a lighter financial burden.^[6]^ Ravi et al showed that among other risk factors, the surgeon's yearly volume can also affect the rate of PPHFx. In their study, for the patients operated on by surgeons who had ≤35 procedures per year, the risks for complications increased by more than 40%.^[10]^

Considerable attention has been given to characteristics that affect the risk of PPHFx. Published studies revealed that most important risk factors were patient demographics (age, sex, and body mass index), and clinical characteristics (preoperative diagnosis, comorbidities, medical/reoperation history). PPHFx is complications which not only lead to both functional and psychological impacts on patients, but also cause financial burden for the patients and healthcare system.^[5,6,11,12]^ Though there were some studies done on PPHFx showing financial burden to be $24,831 for the average hospital length of stay of 6.3 ± 8.8 days,^[13]^ €26,436 (equivalent to $29,995) for the average length of stay of 21.0 days,^[14]^ £33,789 (equivalent to $42,630) for >30 days of hospital stay^[15]^ there is still insufficient or no data to clearly characterize the burden imposed separately by intraoperative and early postoperative (≤90 days) PPHFx in terms of medical resource utilization and costs.

This study aimed to assess the burden associated with intraoperative and early postoperative (≤90 days) PPHFx following primary THA on 90-day and 1-year health care resource utilization and costs in the Medicare population.

## 2 Methods

### 2.1 Data sources

Medicare is one of the largest health insurance programs in the United States, providing coverage to persons 65 years or older and persons younger than 65 years who have end-stage renal disease (ESRD) or who are disabled. This study used data from the United States Centers for Medicare and Medicaid Services (CMS) Standard Analytic File. The data included Medicare Part A and Part B claims which captured Fee-for-Service services. Medicare Advantage Patients were not captured. Medicare Part A captures inpatient hospital visits and related claims including diagnosis (The International Classification of Diseases, Ninth/Tenth Revision, Clinical Modification, ICD-9-CM, ICD-10-CM), procedures, Medicare Severity Diagnosis Related Group, dates of service, hospital provider number, and beneficiary demographic information. Medicare Part B is available for institutional outpatient providers only. Examples of institutional outpatient providers include hospital outpatient departments, rural health clinics, renal dialysis facilities, outpatient rehabilitation facilities, comprehensive outpatient rehabilitation facilities, and community mental health centers. Available data elements include diagnosis (ICD-9 and ICD-10), Healthcare Common Procedure Coding System, dates of service, outpatient provider number, revenue center codes, and beneficiary demographic information. Once an individual enrolls in Medicare they generally remain enrolled until death; hence, this database is ideal for longitudinal studies.

The use of CMS database are Health Insurance Portability and Accountability Act compliant and thus exempt from institutional review board approvals.

### 2.2 Study population

This study used a retrospective longitudinal cohort design identifying patients aged 65+ with a claim for primary THA and diagnosis of hip osteoarthritis. The “Index” date for each patient was defined as the day of discharge from initial THA. All patients were required to have continuous availability of data for at least 365 days before THA and 90 days after the index date. Three cohorts were identified

(1) Patients with intraoperative PPHFx identified between 2010 and 2016. Intraoperative PPHFx was defined as a combination of codes for THA and fixation or arthroplasty of hip-related procedure during same hospitalization.
(2) Patients with postoperative PPHFx identified between 2010 and 2016. Postoperative PPHFx was defined as a combination of codes for periprosthetic fractures (ICD-9-CM, 996.44) and other hip-related fracture diagnosis; or using ICD-10-CM PPHFx codes. Only patients with postoperative PPHFx within 90 days of the index date were included.
(3) Patients without PPHFx anytime during the study period, the control group.

Patients were excluded if they

(1) had a non-Medicare primary payer,
(2) were eligible for Medicare due to ESRD, or
(3) died anytime during the 90 days after the index discharge.

### 2.3 Outcome variables

Ninety (90) and 365-day direct medical resource utilization were analyzed. These included distinct visits (percentage of patients with any visits as yes/no and mean [standard deviation] number of days/visits) and Medicare payment amounts across service types and settings of care (excluding retail pharmacy): skilled nursing facility (SNF), outpatient hospital department, inpatient rehab facility, readmissions.

### 2.4 Matching

A multi-step approach to maximize similarity between patients with and without PPHFx was used. Patients in the PPHFx cohort were matched 1:1 with the control cohort, using both direct and propensity score matching. Patient were first matched directly on the year of THA, surgeon and hospital and then further matched using the propensity scores based on the following covariates: age, gender, race, body mass index, obesity, morbid obesity, congestive heart failure, osteoporosis, opioid dependence or abuse, diabetes, tobacco use and Charlson comorbidity index (CCI). The propensity score matching method involved nearest neighbor technique with calipers of width equal to 0.2 of the pooled standard deviation of the logit of the propensity score.

### 2.5 Data analysis

Frequency counts and proportions were provided for categorical variables. Means and standard deviations were provided for continuous variables. Standardized differences (Std Diff) and tests of significance were used to compare the differences between the cohorts, with and without PPHFx, for the patient demographic and clinical characteristics before and after matching. A Std Diff below 0.1 was concluded to indicate a negligible difference between compared groups for each measure. Health care resource utilization and costs over 90 days and 365 days were compared using Wilcoxon signed rank test for continuous variables and McNemar test for categorical variable. The costs were not adjusted for inflation as Medicare payments have shown minimal changes to inflation.

## 3 Results

### 3.1 Pre-matched cohorts: Baseline characteristics

The study included 2976 patients with intraoperative, 1479 patients with postoperative PPHFx and 473,602 patients without PPHFx. Baseline (pre-match) patient demographic and clinical characteristics for the intraoperative and postoperative PPHFx cohorts versus pre-matched control cohort are presented in Tables 1 and 2. The mean age of the intraoperative and postoperative cohorts were 76.4 (±7.0) years and 75.1 (±6.5) years, respectively. There were greater proportions of women in intraoperative (75.9%, Std Diff = 0.30) and postoperative (77.3%, Std Diff = 0.32) cohorts as compared to the pre-matched control cohort (62.4%). Patients in the intraoperative PPHFx cohort were more likely to have osteoporosis than the control cohort (36.1% vs 19.8%, Std Diff = 0.37). Patients in the postoperative PPHFx cohort were more likely to have obesity (25.3% vs 18.2%, Std Diff = 0.17), morbid obesity (8.8% vs 5.3%, Std Diff = 0.14), osteoporosis (27.0% vs 19.8%, Std Diff = 0.17), and tobacco use (32.9% vs 23.4%, Std Diff = 0.21) than the control cohort. In addition, patients with intraoperative (41.7% vs 34.2%, Std Diff = 0.17) or postoperative (37.7% vs 34.2%, Std Diff = 0.11) PPHFx were more likely than control cohort to have a CCI score greater than 1.

**Table 1:**
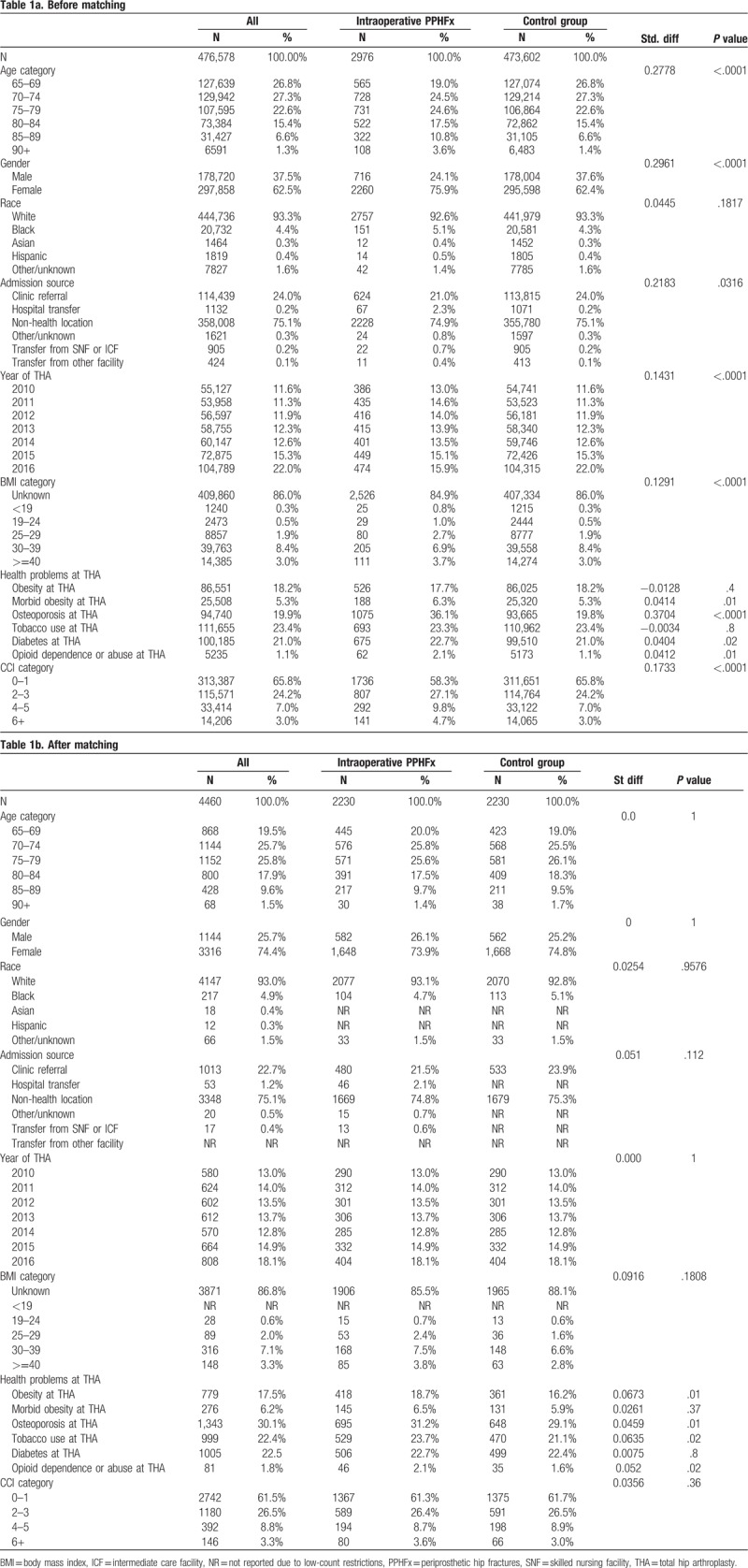
Baseline patient characteristics before (Table 1a) and after (Table 1b) matching for patients with intraoperative PPHFx and control group (patients with THA and no PPHFx).

**Table 2:**
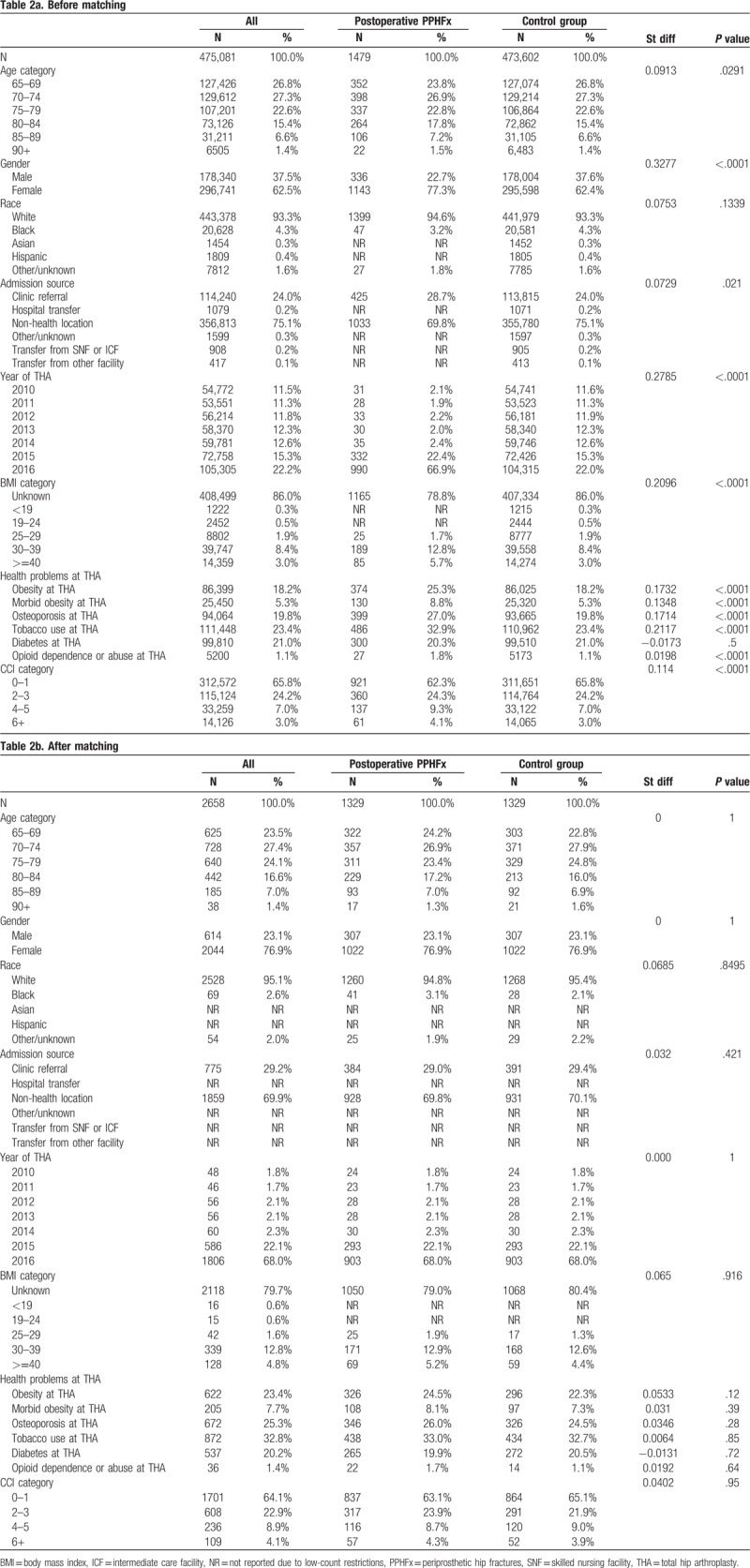
Baseline patient characteristics before (Table 2a) and after (Table 2b) matching for patients with early postoperative PPHFx and control group (patients with THA and no PPHFx).

### 3.2 Matched cohorts: Baseline characteristics

After applying direct and propensity-score matching techniques, 4460 patients (2230 intraoperative and 2230 controls) and 2658 patients (1329 postoperative and 1329 controls), remained available for comparative analysis for intraoperative and postoperative PPHFx, respectively. No significant between-group differences in baseline patient demographic and clinical characteristics were observed for these matched cohorts. Patients with intraoperative PPHFx were similar to control patients with respect to mean age (75.7 ± 6.5 vs 75.9 ± 6.5, Std Diff = 0.03), gender (73.9% females vs 74.8% females, Std Diff = −0.02), race (93.1% Whites vs 92.8% Whites, Std Diff = 0.03), and various comorbid conditions and CCI score (all Std Diff < 0.1, indicating negligible differences). Similarly, patients with postoperative PPHFx were similar to control patients with respect to mean age (75.0 ± 6.4 vs 79.9 ± 6.3, Std Diff = 0.01), gender (76.9% females vs 76.9% females, Std Diff = 0.00), race (94.8% Whites vs 95.4% Whites, Std Diff = 0.07), and various comorbid conditions and CCI score (all Std Diff < 0.1, indicating negligible differences).

### 3.3 Matched cohorts- direct medical resource utilization and payments

Tables 3–6 depict the information about the patient utilization, days of service and Medicare claim payments associated with treatment of intraoperative and postoperative PPHFx patients for 90- and 365-day periods after hospitalization.

**Table 3:**
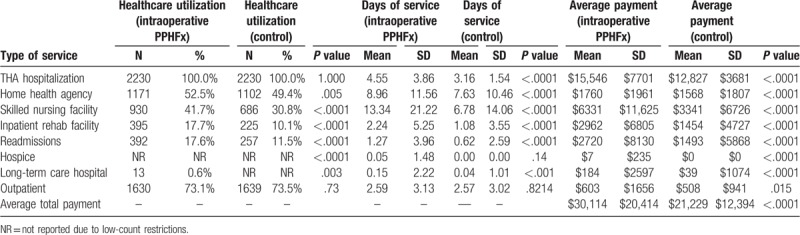
Healthcare utilization, days of service, and Medicare payments associated with treating intraoperative periprosthetic hip fracture over a 90-d period after discharge from hospitalization for THA (intraoperative PPHFx and control groups) (N = 4460).

**Table 4:**
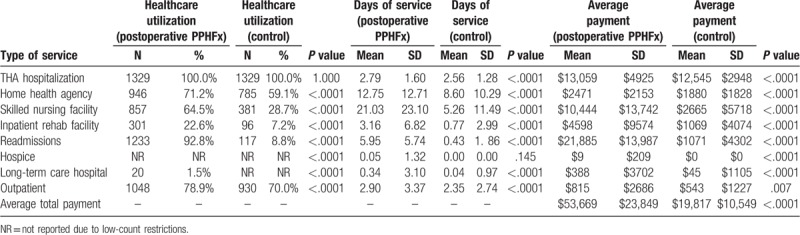
Healthcare utilization, days of service, and Medicare claim payments associated with treating postoperative periprosthetic hip fracture patients over a 90-d period after discharge from hospitalization for THA (postoperative PPHFx and control groups) (N = 2658).

**Table 5:**
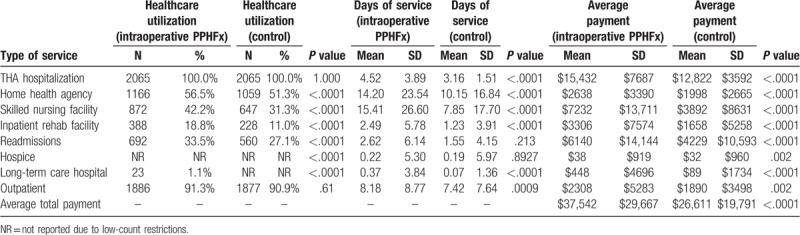
Healthcare utilization, days of service, and Medicare claim payments associated with treating intraoperative periprosthetic hip fracture patients over a 365-d period after discharge from hospitalization for THA (intraoperative PPHFx and control groups) (N = 4130).

**Table 6:**
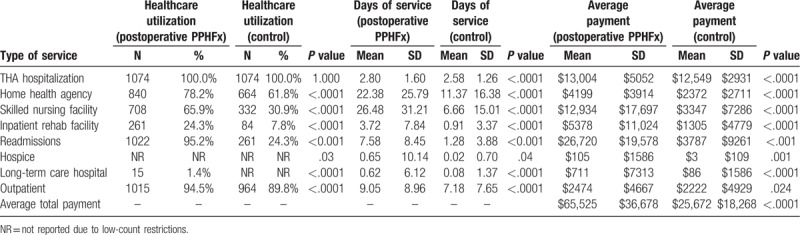
Healthcare utilization, days of service, and Medicare claim payments associated with treating postoperative periprosthetic hip fracture patients over a 365-d period after discharge from hospitalization for THA (postoperative PPHFx and control groups) (N = 2148).

Patients with intraoperative PPHFx had significantly higher hospital length of stay for the index THA procedure which involved fixing the intraoperative fracture when compared to matched patients in the control group (4.55 ± 3.86 days vs 3.16 ± 1.54 days, *P* < .0001) (Table 3).

Utilization of any 90-day post-acute services (Table 3) was also significantly higher among patients in the intraoperative cohort versus those in the control cohort: SNF (41.7% vs 30.8%, *P* < .0001), inpatient rehabilitation facility (17.7% vs 10.1%, *P* < .0001), and readmissions (17.6% vs 11.5%, *P* < .0001). The length of stay in each of these settings was also significantly higher in the intraoperative PPHFx cohort: SNF (13.34 ± 21.22 days vs 6.78 ± 14.06 days, *P* < .0001), inpatient rehabilitation facility (2.24 ± 5.25 days vs 1.08 ± 3.55 days, *P* < .0001), and readmissions (1.27 ± 3.96 days vs 0.62 ± 2.59 days, *P* < .0001).

Similarly, patients with early postoperative PPHFx had significantly higher hospital length of stay for the index THA procedure when compared to matched patients in the control group (2.79 ± 1.60 days vs 2.56 ± 1.28 days, *P* < .0001) (Table 4).

Utilization of any 90-day post-acute services (Table 4) was also significantly higher among patients in the early postoperative PPHFx cohort versus those in the control cohort: SNF (64.5% vs 28.7%, *P* < .0001), inpatient rehabilitation facility (22.6% vs 7.2%, *P* < .0001), and readmissions (92.8% vs 8.8%, *P* < .0001). The length of stay in each of these settings were also significantly higher in the early postoperative PPHFx cohort: SNF (21.03 ± 23.10 days vs 5.26 ± 11.49 days, *P* < .0001), inpatient rehabilitation facility (3.16 ± 6.82 days vs 0.77 ± 2.99 days, *P* < .0001), and readmissions (5.95 ± 5.74 days vs 0.43 ± 1.86 days, *P* < .0001).

All trends were similar in the 365-day follow up for both intraoperative and early postoperative PPHFx cohorts (Tables 5 and 6)

Mean 90-day total all-cause payments were significantly higher for patients with intraoperative cohort versus control patients ($30,114 vs $21,229, *P* < .0001) (Table 3). These included significantly higher payments for index hospitalizations ($15,546 vs $12,827, *P* < .0001), SNF ($6331 vs $3341, *P* < .0001), inpatient rehabilitation facility ($2962 vs $1454, *P* < .0001), and readmissions ($2720 vs $ 1493, *P* < .0001). Similarly, mean 365-day total all-cause payments were significantly higher for patients with intraoperative cohort versus control patients ($37,542 vs $26,611, *P* < .0001) (Table 5).

For patients with early postoperative PPHFx the mean 90-day total all-cause payments were significantly higher versus control patients ($53,669 vs $ 19,817, *P* < .0001) (Table 4). These included significantly higher payments for index hospitalizations ($13,059 vs $12,545, *P* < .0001), SNF ($10,444 vs $2665, *P* < .0001), inpatient rehabilitation facility ($4598 vs $1069, *P* < .0001), and readmissions ($21,885 vs $ 1071, *P* < .0001). Similarly, mean 365-day total all-cause payments were significantly higher for patients with early postoperative cohort versus control patients ($65,525 vs $25,672, *P* < .0001) (Table 6).

## 4 Discussion

In this study, we assessed the impact of PPHFx during or after THA on 90-day and 1-year costs in the Medicare population. The patients with intraoperative PPHFx had economically important and statistically significantly higher resource utilization when compared to patients without PPHFx. Similarly, patients with postoperative PPHFx had statistically significantly higher resource utilization and payments than patients without PPHFx. These differences were observed during the 90-day follow up and continued over the 1-year period as well.

Thillemann et al found that intraoperative PPHFx increase the risk of revision during the first 6 months postoperatively. The overall cumulative revision rate was 3.4% within 6 months postoperatively for patients with intraoperative PPHFx versus 0.9% for patients without intraoperative PPHFx fractures (*P* < .001). The authors also found a significant increase in hospital stay for those patients with intraoperative PPHFx (11 days to 13 days *P* < .001)^[16]^ Although our research did not evaluate the rate of revision after intraoperative PPHFx, we have evaluated the overall readmission rates, of which some would be for revision, over the 90-day and 1-year period after the index operation. We found that patients with intraoperative PPHFx had higher rate of all-cause readmissions (17.6% vs 11.5% at 90-day; 33.5% vs 27.1% at 1-year) as compared to patients without intraoperative fractures.

Nishihara et al found that patients with intraoperative PPHFx achieved significantly lower ability to walk with cane within 13 days after THA as compared to the group without PPHFx (*P* < .05).^[17]^ Intraoperative PPHFx can result in a prolonged surgery and delay in full weight bearing after surgery. Consequently, patients with intraoperative PPHFx may need higher post-acute care after THA as compared to patients without. Our research shows statistically significantly higher percent of utilization and days of service for SNF (41.7% vs 30.8%; 13.3 days vs 6.8 days) and inpatient rehab facility (17.7% vs 10.1%; 2.2 days vs 1.1 days) in the 90-day follow-up after THA for patients with intraoperative PPHFx as compared to patients without PPHFx.

Studies have shown approaches to mitigate the risk of intraoperative PPHFx. Nishihara et al describe a series of patients that were hand-rasped vs a series of patients that were robotically milled. In the robotically milled group (n = 78) there were no reported intraoperative femoral fractures compared with the hand-rasped patients, where 5/78 procedures resulted in intraoperative femoral fractures.^[17]^ Another method that might benefit patients relates to an adjustment of “hammering force” during stem impaction. An experimental in-vitro study by Sakai et al used finite element analysis to demonstrate that 2 hammer strikes were sufficient to seat the femoral stem, with further hammer strikes of similar force having little effect on the stem displacement but instead increasing the risk of microfracture due to stress concentration in the medial calcar region.^[18]^

Early postoperative PPHFx as defined in this study were those fractures occurring within 90 days post-THA. These fractures sometimes may be linked to an undiagnosed intraoperative PPHFx, which probably is an important risk factor for predicting an early postoperative fracture. Our study showed statistically significantly higher healthcare utilization and costs in patients with postoperative PPHFx as compared to patients without PPHFx. In addition, a numerical higher difference in the costs was observed with postoperative than intraoperative PPHFx as compared to patients without PPHFx indicating serious clinical implications, including the treatment and outcome with postoperative PPHFx.

Our study showed that a majority of patients with PPHFx – higher than 75.0% in both intraoperative and post-operative treatment cohorts – were women. This may be due to higher prevalence of osteoporosis in women and differences in bone structure. Other studies in past also had results confirming this finding and showed higher risk of PPHFx in women.^[19]^ Jasvinder et al showed that gender was significantly associated with the higher risk of PPHFx within 1 year with hazard ratio 2.61 (95% confidence interval, 1.68, 4.05). The same study showed that CCI was significantly associated with higher risk of PPHFx both <1 year and >1 year.^[20,21]^ This finding also is consistent with our findings, as our study has also shown statistically significant difference between PPHFx (both intraoperative and postoperative) and control groups in terms of CCI category in the unmatched cohorts.

Our study has important strengths and limitations. In particular, it is unique for the use of rigorous patient-matching techniques spanning patient demographic and clinical characteristics and exact matching on the hierarchical variables like surgeon and hospital that influence outcomes. An additional strength arises from the large sample size which provides the study with adequate power. This was also the first study to our knowledge that characterized data for different settings of care separately for intraoperative and postoperative PPHFx following THA. Limitations of the study include use of combination of codes from the claims data to define intraoperative and postoperative PPHFx, which may result in underreporting on the incidence of PPHFx. However, another reason for underreporting intraoperative fractures, not related to the study design, is that they are not recognized during surgery. Schwartz et al reported that half of their intraoperative PPHFx were not detected during surgery but diagnosed on postoperative radiographs.^[22]^ This study is further limited by excluding pharmaceuticals, durable medical equipment, and indirect costs. The analysis is limited to a Medicare fee-for-service population, and therefore cannot draw conclusions about results for patients with other payers, including commercial insurers. As with any retrospective study, unmeasured factors (eg, patient expectations) could not be matched and may have contributed to between-group differences.

## 5 Conclusion

After direct and propensity score matching it was found that the patients with PPHFx during or following primary THA had significantly increased healthcare utilization and costs during the 90 days and 1-year follow-up than patients without PPHFx for both intraoperative and early postoperative PPHFx. Approaches that could mitigate the risk of PPHFx are warranted to reduce the healthcare burden.

## Author contributions

**Conceptualization:** Abhishek S. Chitnis, Jack Mantel, Mollie Vanderkarr, Matthew Putnam, Chantal E. Holy, Joshua Bridgens.

**Data curation:** Jill Ruppenkamp.

**Formal analysis:** Abhishek S. Chitnis, Jill Ruppenkamp.

**Funding acquisition:** Jack Mantel, Mollie Vanderkarr.

**Methodology:** Abhishek S. Chitnis, Jack Mantel, Matthew Putnam, Jill Ruppenkamp, Chantal E. Holy, Joshua Bridgens.

**Project administration:** Chantal E. Holy.

**Resources:** Jack Mantel, Matthew Putnam, Chantal E. Holy.

**Supervision:** Abhishek S. Chitnis, Jack Mantel, Mollie Vanderkarr, Matthew Putnam, Chantal E. Holy, Joshua Bridgens.

**Validation:** Abhishek S. Chitnis, Chantal E. Holy.

**Writing – original draft:** Abhishek S. Chitnis, Jack Mantel, Mollie Vanderkarr, Matthew Putnam, Jill Ruppenkamp, Chantal E. Holy, Joshua Bridgens.

**Writing – review and editing:** Abhishek S. Chitnis, Jack Mantel, Mollie Vanderkarr, Matthew Putnam, Jill Ruppenkamp, Chantal E. Holy, Joshua Bridgens.
